# Abstract of the Proceedings of the Buffalo Medical Association

**Published:** 1876-08

**Authors:** E. N. Brush

**Affiliations:** Secy.


ART. II.—*Abstract of the Proceedings of the Buffalo Medical Association*, *June* 16, 1876. Dr. E. N. Brush, Secy.

The President Dr. Wyckoff, in the chair. Present—Drs. Rochester, Abbott, White, Fowler, Brecht, O'Brien, Hopkins, Phelps,

Bielby, Howe, Strong, Miner, Wetmore, Hebenstreit, W. W. Miner, Samo and Boysen. Dr. Baker by invitation.

The minutes were read and approved.

The application of Dr. F. A. Burghardt was taken from the table and he was elected a member of the Association.

Dr. Abbot said that he had lately had several cases of inflammation, both acute and chronic, of the eustachian tube and middle ear, in which the treatment which he had pursued, had met with such favorable results that he thought it of sufficient interest to report to the association. He had employed the vapor of hot water, throwing it into the eustachian tube, combining with this, when necessary, an injection of sulphate of zinc, two to five grains to the ounce.

The vapor is forced into the tube and middle ear by forcing air through a flask of water, in a water bath.

He reported the following case:

A reporter on one of the daily papers came to him a few days since, suffering with deafness in the right ear. The watch could only be heard at a distance of two and one-half inches, and ordinary conversation could not be heard at all on that side. The vapor of hot water was employed as described. The patient came every other day until he had four treatments, then, after an intermission of a week, he came again. In that time the hearing distance had increased to two feet. The zinc was used but twice, the improvement was so rapid under the vapor of water that the zinc was not found necessary.

He had another case recently, which came to him complaining of an intolerable itching and accumulation in the external meatus. He supposed it to be a case of aspergillus fungus. On examination of the deposit under the microscope, it was found to be this vegetable parasite, which causes extreme itching of the meatus, and frequently, deafness.

Warm water and nitrate of silver gr. xl. to ℥ j., were applied, affecting a permanent cure.

He also reported a case which had been brought to him for the removal of a glass bead from the meatus. With the help of another physician, the family physician had made, under chloroform, attempts to remove it. The measures undertaken had evidently been of a violent nature as the meatus was filled with blood, and its lining red and swollen. The ear was carefully syringed and a few drops of sweet oil directed to be used. In two or three days the bead dropped out. The case was reported to illustrate the inadvisability and danger of using force in removing foreign bodies from the meatus.

Dr. Hopkins said that he would like to ask Dr. Abbot if the natural history of the disease in the case which he first reported was sufficiently well defined, by statistics, to warrant him in saying that his measures were curative, or whether the cure was simply a natural termination of the catarrhal trouble.

Dr. Abbot replied that he could not say as to statistics, but that the improvement in the condition of the patient's hearing, warranted him in thinking that the treatment which he had adopted, led to the recovery.

Dr. Miner asked if simple inflation of the eustachian tube by warm air without moisture would not have produced equally good results.

Dr. Rochester inquired as to the use of the eustachian catheter. He said that in his younger days it was frequently used. Heard a distinguished aural surgeon, now living, say that he had no doubt that many cases of deafness were caused by the injudicious employment of the eustachian catheter. Would ask as a matter of information, whether the catheter was as much used at the present day as formerly. It seemed to him that warm water or other substances thrown into the eustachian tube would be liable to produce irritation.

Dr. Abbott said that he thought the cases were now more carefully selected, that the catheter was not employed as promiscuously as formerly. That in his own practice, where the apparatus of Politzer was used with benefit, he did not employ the catheter, but where the apparatus did not answer the demands of the case, he employed the catheter. He used a hard rubber catheter in preference to a silver one. It should be used with delicacy and care.

Dr. Strong asked if it was always harmless to inject into the middle ear through the eustachian tube.

Dr. Abbott replied that where the vapor of warm water was used he had never seen any unfavorable results or with mild astringent solutions. It could scarcely be called an injection with the apparatus which he used, but a small amount of fluid could get into the ear.

Dr. Howe said that in regard to Dr. Rochester's question, he had no doubt but what the catheter could be and had been abused. The old method of holding the nostrils shut and swallowing, or the employments of the apparatus of Politzer, acted equally on both ears. With the catheter the medicament could be passed directly to one side, which was one advantage. In another class of cases the secretion is tenacious and sticky, and the tube could not be inflated by the ordinary methods. In reference to Dr. Abbott's method by hot water he found that it did not suit all cases. He had also found that some patients objected to being scalded by hot water.

Dr. Abbott said that he had never had a patient complain of heat. The apparatus which he used prevented the injection being used too hot.

Dr. Hopkins said that he would like to report a case of tape-worm, ccurring in a child of six years. He reported it as being somewhat unusual to find this parasite in so young a child.

He gave half a drachm of kousso, followed by a cathartic, but it was entirely inert.

The fluid extract of the male fern was then used, half a drachm being given in two doses, which was followed by the expulsion of the tape-worm entire.

Dr. Rochester said that he had seen a child of two years, which was relieved of a tape-worm by kousso. The explanation of its failure would, perhaps, be that the article was not of good quality. As to the origin of tape-worm Cobbold speaks of the tinea of mutton, pork and beef.

Dr. White asked if Dr. Rochester was positive that the tinea of mutton was propagated in the human family. He said that he had seen a statement made to the contrary.

Dr. Rochester replied that Cobbold and other authorities affirm that it is.

Dr. Wyckoff suggested that the great frequence of tape-worm might probably be explained by the prevalence of eating beef in an under-done condition.

Dr. Wetmore asked if any gentleman had found two tape-worms in the same individual. He had in his office a tape-worm forty five feet in length, with the head attached. Two weeks after its removal another began to pass which was finally brought away entire, by the use of turpentine.

Dr. Wyckoff called Dr. O'Brien to the chair, and reported a case of chronic cystitis in a lady aged forty-eight.

The patient had suffered for several years with cystitis, and had been the victim of various measures for its relief, but to no effect. The bladder was very irritable, the patient finding it necessary to void urine very frequently. On April 20th he dilated the urethra to such an extent that he could introduce the thumb. This was followed by marked relief; the irritability of the bladder was in a measure relieved so that it was not necessary to void urine as frequently as formerly.

On the twenty-third of May he again dilated the urethra so that he could introduce two fingers, he then introduced a speculum that was seven-eighths of an inch in diameter. No incontinence of urine followed, the patient was able immediately afterward to retain the urine, and since then has been able to do so for three or four hours at time. The irritability of the bladder is decreasing and further improvement is looked for.

Dr. Miner said physicians would be surprised, who had not witnessed the operation, at the extent to which the female urethra could be dilated without producing incontinence. This was also the case with the neck of the bladder and prostatic urethra in young males. He had removed from a boy, aged twelve, by the median operation, a calculus, which when grasped in the forceps as it passed through the neck of the bladder, measured four and one-half inches in circumference. This operation was only followed by incontinence of urine for a few days, the boy rapidly gaining control of his bladder as he convalesced. He had on several occasions gone beyond the limit to which authors state that the neck of the bladder and prostatic urethra could be dilated, but had never met with any unpleasant symptoms resulting from the over-dilation of the urethra.

DR. WHITE remarked that the case reported by Dr. Wyckoff was very interesting; he could not exactly understand, however, how the simple dilation of the urethra could relieve cystitis, the bladder still remaining continent. The only hypothesis upon which he could explain it was that the irritability was due to spasm of the sphincter vesicæ, which the stretching overcame. He had in New York, some few years since, assisted Dr. Sims in making an operation to establish a vesico-vaginal fistula, to relieve a young woman of cystitis, the object being to prevent the accumulation of urine, and thus give the bladder perfect rest. Dr. White expressed his preference in dilating the urethra for the removal of foreign bodies, or for other purposes, to the employment of a bivalve speculum, by means of which the urethra could gradually be stretched.

DR. WYCKOFF said that he had used his finger entirely, and that by its employment the surgeon could best estimate the extent to which the dilation could be carried without danger of rupturing the walls of the urethra.

DR. WETMORE said that but a few days ago he had the pleasure of listening to a clinical lecture by Prof. Gross, on this very subject, he has great confidence in the employment of injections and the administration of Potassium Bromide and Gelsemium.

He first washes out the bladder with an ounce of water, then with two, and so on, gradually distending its walls. He was very particular in the manner in which the injections were used, it being his object to imitate the gradual distention of the bladder by urine as nearly as possible, and for this reason he began with a small amount of fluid at first and made his injection very slowly.

The Secretary presented a communication from the Boston Society of Civil Engineers, in reference to the metric system of weights and measures. He was directed to acknowledge the communication and inform the committee that some action would probably be taken on the subject at the coming International Medical Congress.

An amendment to the Constitution and By-Laws presented at the April meeting, was brought up and after some discussion, was made the special order of business at the next meeting.

DR. WHITE moved that when the meeting adjourned, it do so to meet on Wednesday evening, July 5th.—Carried.

The meeting was then on motion declared adjourned.

